# Hydrogen Gas Is Involved in Auxin-Induced Lateral Root Formation by Modulating Nitric Oxide Synthesis

**DOI:** 10.3390/ijms18102084

**Published:** 2017-10-03

**Authors:** Zeyu Cao, Xingliang Duan, Ping Yao, Weiti Cui, Dan Cheng, Jing Zhang, Qijiang Jin, Jun Chen, Tianshan Dai, Wenbiao Shen

**Affiliations:** 1 College of Life Sciences, Laboratory Center of Life Sciences, Nanjing Agricultural University, Nanjing 210095, China; 2017116113@njau.edu.cn (Z.C.); 2016216026@njau.edu.cn (X.D.); 2016116112@njau.edu.cn (P.Y.); wtcui@njau.edu.cn (W.C.); softcheng@njau.edu.cn (D.C.); zhangjingjhc@sjtu.edu.cn (J.Z.); jqj@njau.edu.cn (Q.J.); 2 Wuhan Shizhen Water Structure Research Institute Co., Ltd., Wuhan 430200, China; chenjun@shizhensss.com; 3 Xinjiang Hongsheng Kangtong Biotechnology Co., Ltd., Xinjiang 830022, China; tsdai@shizhensss.com

**Keywords:** H_2_, auxin, lateral root formation, NO, nitrate reductase

## Abstract

Metabolism of molecular hydrogen (H_2_) in bacteria and algae has been widely studied, and it has attracted increasing attention in the context of animals and plants. However, the role of endogenous H_2_ in lateral root (LR) formation is still unclear. Here, our results showed that H_2_-induced lateral root formation is a universal event. Naphthalene-1-acetic acid (NAA; the auxin analog) was able to trigger endogenous H_2_ production in tomato seedlings, and a contrasting response was observed in the presence of *N*-1-naphthyphthalamic acid (NPA), an auxin transport inhibitor. NPA-triggered the inhibition of H_2_ production and thereafter lateral root development was rescued by exogenously applied H_2_. Detection of endogenous nitric oxide (NO) by the specific probe 4-amino-5-methylamino-2′,7′-difluorofluorescein diacetate (DAF-FM DA) and electron paramagnetic resonance (EPR) analyses revealed that the NO level was increased in both NAA- and H_2_-treated tomato seedlings. Furthermore, NO production and thereafter LR formation induced by auxin and H_2_ were prevented by 2-4-carboxyphenyl-4,4,5,5-tetramethylimidazoline-1-oxyl-3-oxide (cPTIO; a specific scavenger of NO) and the inhibitor of nitrate reductase (NR; an important NO synthetic enzyme). Molecular evidence confirmed that some representative NO-targeted cell cycle regulatory genes were also induced by H_2_, but was impaired by the removal of endogenous NO. Genetic evidence suggested that in the presence of H_2_, Arabidopsis mutants *nia2* (in particular) and *nia1* (two nitrate reductases (NR)-defective mutants) exhibited defects in lateral root length. Together, these results demonstrated that auxin-induced H_2_ production was associated with lateral root formation, at least partially via a NR-dependent NO synthesis.

## 1. Introduction

In higher plants, the formation of lateral root (LR) is influenced by phytohormones and a wide range of environmental cues, including water availability, nutrients, and abiotic stress [1,2,3]. Previous studies suggested that auxin regulates and coordinates both lateral root founder cell divisions and polarity during lateral root initiation [3,4]. Genetic and molecular evidence suggested that auxin modulates several cell cycle regulatory genes responsible for lateral root formation, such as *CYCA2;1*, *CYCA3;1*, *CYCD3;1*, *CDKA1*, and *KRP2* in Arabidopsis [1,5]. However, *N*-1-naphthylphthalamic acid (NPA), an effective blocker of auxin polar transport, significantly blocked auxin-induced lateral root development [6]. Ample evidence further revealed that nitric oxide (NO) is a ubiquitous and free radical gas that regulates a wide range of physiological processes in plants, and the important roles of NO in auxin-triggered lateral root formation [7], root hair development [8], and adventitious rooting [9], were discovered. Despite these discoveries, the understanding of the mechanisms of NO biosynthesis in plants is still incomplete. So far, two enzymes mainly involved in NO production were suggested, after the experiments with the inhibitor tests by the removal of endogenous NO, including the application of tungstate (a nitrate reductase (NR) inhibitor) and *N*^G^-nitro-l-arginine methyl ester (a nitric oxide synthetase (NOS)-like protein inhibitor) [10,11].

Hydrogen gas (H_2_) is the lightest and most affluent element in the world, constituting about 75% of universe’s elemental mass. The metabolism of H_2_ by bacteria, green algae, and higher plants, has been reported for many years [12]. Previous studies discovered that H_2_ is a possible anti-oxidant and anti-inflammatory agent with promising application in clinical treatment [13,14]. For instance, H_2_-rich saline protected lung tissue against injury caused by hypertoxic exposure in rats [15]. Similar to the beneficial roles in animals, H_2_ has emerged as an important gaseous molecule under abiotic stress and several physiological processes in plants. For example, H_2_ alleviated Al-induced inhibition of alfalfa root elongation by decreasing NO production in alfalfa seedlings [16]. The involvement of H_2_ in the promotion of cucumber adventitious root formation was also discovered [17]. Although H_2_ and NO were respectively suggested to be required for root architecture, the potential interaction between H_2_ and NO during lateral formation is unclear.

To answer this scientific question, we found herein that H_2_ is involved in auxin-induced lateral root development in tomato seedlings, mimicking the responses of exogenously applied 1-naphthylacetic acid (NAA; the auxin analog). Auxin-induced H_2_ production is a very interesting observation. Molecular and pharmacological approaches further revealed that NO may operate downstream of H_2_ promoting lateral root formation by the modulation of cell cycle regulatory genes. By using the genetic and pharmacological approaches, our results indicated the role of nitrate reductase (NR) in H_2_-induced Arabidopsis lateral root formation. Our results contribute new insight to our understanding auxin and NO signaling governing root organogenesis in plants.

## 2. Results

### 2.1. Exogenous Hydrogen Gas (H_2_)-Induced Lateral Root Formation in A Dose-Dependent Manner

**Figure 1:**
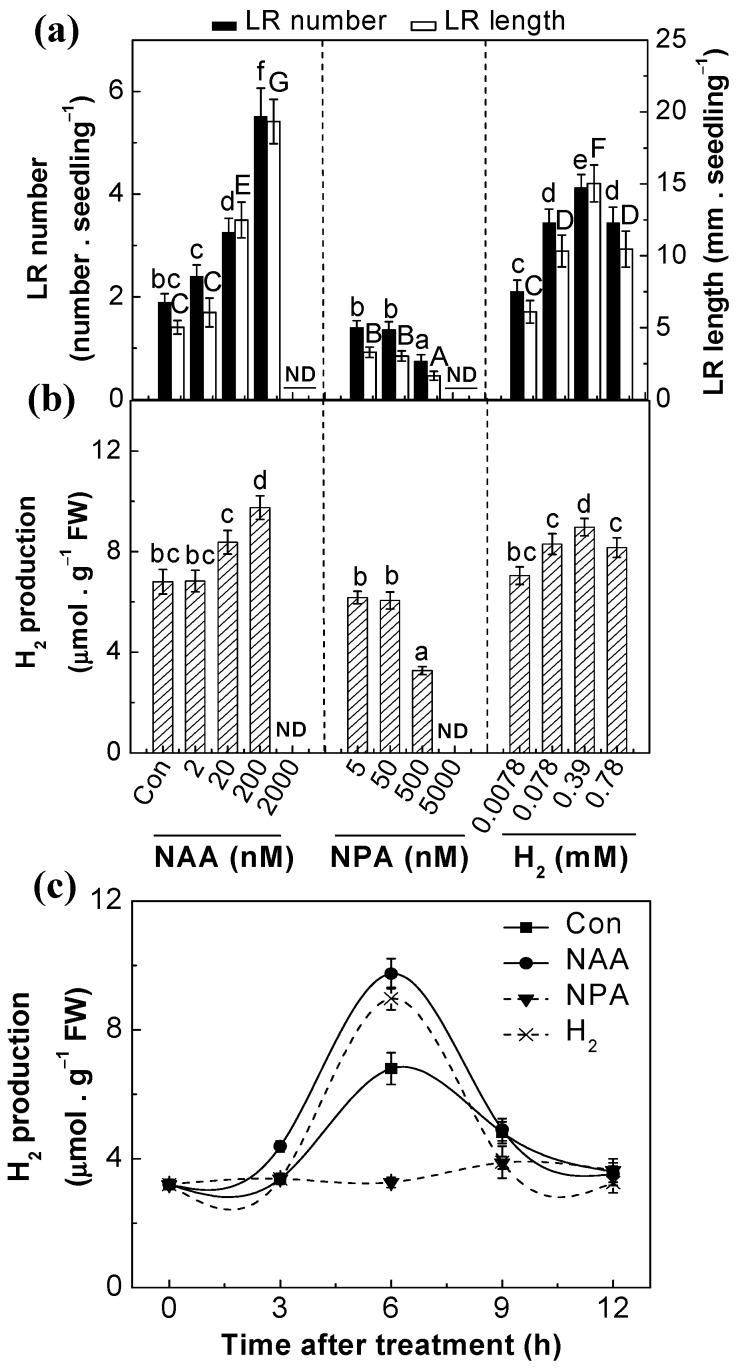
Effects of 1-naphthylacetic acid (NAA), *N*-1-naphthylphthalamic acid (NPA), and H_2_ on lateral root formation and H_2_ release. 3-day-old tomato seedlings were incubated with solutions containing various concentrations of NAA, NPA, and H_2_. (**a**) The number of emerged lateral roots (LRs) (>1 mm) per seedling and LR length were calculated after 3-day of treatments (*n* = 60); (**b**) The H_2_ production in roots was detected after 6-h treatment (*n* = 5); (**c**) Time-course of H_2_ production in response to 200 nM NAA, 500 nM NPA, and 0.39 mM H_2_ (*n* = 5). Distilled water was used for the control (Con) treatment. Data are the means ± SE of three independent experiments with at least three replicates for each. Within each set of experiments, bars denoted by the same letter did not differ significantly at the *p* < 0.05 level according to Duncan’s multiple range test.

To verify whether H_2_ had any effects on the lateral root formation in plants, tomato seedlings were incubated with solutions containing different concentrations of exogenous H_2_ (0.0078, 0.078, 0.39, and 0.78 mM). Compared to the control samples, treatment with exogenous H_2_ induced lateral root formation in a dose-dependent fashion, with a maximal effect in 0.39 mM H_2_ (Figure 1a). For example, it was observed that compared to the control samples, the application of 0.39 mM H_2_ was able to increase lateral root number, length, and primordial number in tomato seedlings by 119%, 273%, and 170%, respectively (Table 1). Thus, 0.39 mM H_2_ was subsequently used in tomato seedlings.

Similar inducing tendencies were observed in different plant species, including *Brassica napus*, *Brassica chinensis*, *Oryza sativa*, and *Zea mays* after treatments with H_2_, and we chose the suitable culture conditions and treated times in the above experiments, for the maximum effect. Therefore, these results revealed that exogenous H_2_-induced lateral root formation is a universal event.

Three-day-old tomato (*Lycopersicon esculentum*) seedlings, 2-day-old rapeseed (*Brassica napus*) seedlings, 3-day-old Chinese cabbage (*Brassica chinensis*), 4-day-old rice (*Oryza sativa*) seedlings, and 7-day-old maize (*Zea mays*) seedlings, were incubated with solutions containing the indicated concentrations of H_2_ for 3, 3, 2, 2 and 1 days. Afterwards, the number of emerged LRs (>1 mm) per seedling and LR length were analyzed. Meanwhile, the number of emerged LRP was also calculated after treatments for 1, 1, 1, 2 days and 6 h, respectively. Distilled water was used for the control (Con) treatment. Mean and SE values were calculated from at least three independent experiments with at least three replicates for each (*n* = 50). Within each set of experiments, dates with asterisks were significantly different with respect to the control at *p* < 0.05 according to *t*-test.

### 2.2. A Possible Link between Auxin and H_2_ in the Induction of Lateral Root Formation

It is well-known that exogenous auxin, including 1-naphthylacetic acid (NAA), could increase lateral root development [5,7]. To investigate the link between auxin and H_2_ in the induction of lateral rooting, 3-day-old tomato seedlings were treated with various concentrations of NAA, *N*-1-naphthylphthalamic acid (NPA; the auxin transport inhibitor), and H_2_. As expected, compared to the control, NAA dose-dependently increased the lateral root number and length, with a maximal response at 200 nM NAA (except 2000 nM; Figure 1a). However, NPA, the auxin transport blocker, obviously suppressed lateral root organogenesis in a dose-dependent manner as well.

Meanwhile, H_2_ level in tomato seedling roots was detected by gas chromatography (GC). Comparatively, endogenous H_2_ production was maximally induced by 200 nM NAA and 0.39 mM H_2_ supplementation for 6 h (Figure 1b,c). Contrasting responses were observed when NPA was exogenously applied. For example, after treatment with 500 nM NPA for 6 h, H_2_ production was obviously reduced by about 52%. Combined with the changes in lateral root formation, these results indicated the possible link between auxin and endogenous H_2_ in the induction of lateral root formation.

### 2.3. H_2_ Was Partly Involved in Auxin-Induced Lateral Root Formation

**Figure 2:**
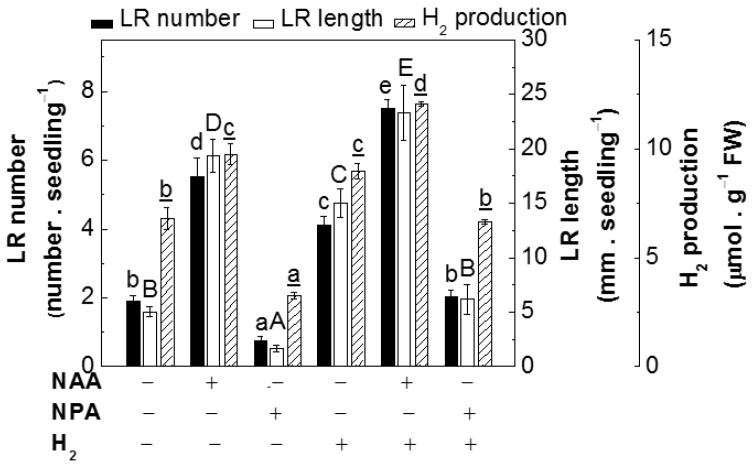
NAA- and NPA-regulated lateral root formation and H_2_ production were regulated by exogenous H_2_. 3-day-old tomato seedlings were incubated with solutions containing 200 nM NAA, 500 nM NPA, and 0.39 mM H_2_, alone or the combination treatments. The number of emerged LRs (>1 mm) per seedling and LR length were calculated after 3-day of treatment (*n* = 60). The H_2_ production in roots was determined after 6-h treatment (*n* = 5). Distilled water was used for the control (Con) treatment. Data are the means ± SE of three independent experiments with at least three replicates for each. Within each set of experiments, bars denoted by the same letter did not differ significantly at the *p* < 0.05 level according to Duncan’s multiple range test.

To confirm the above deduction, tomato seedlings were treated with NPA or NAA in the presence or absence of different concentrations of H_2_. As shown in Figure 2, NPA-inhibited lateral root formation and endogenous H_2_ production in tomato seedling roots were differentially rescued by the supplement of 0.39 mM H_2_, but to lesser degrees than those in 0.39 mM H_2_, or 200 nM NAA-treated alone plants. Meanwhile, additive responses in lateral rooting were observed when NAA was together with 0.39 mM H_2_ (Figure S1). These results suggested that endogenous H_2_ might be, at least partially, involved in lateral root formation promoted by auxin.

### 2.4. Nitric Oxide (NO) Was Involved in H_2_-Promoted Lateral Root Formation

**Figure 3:**
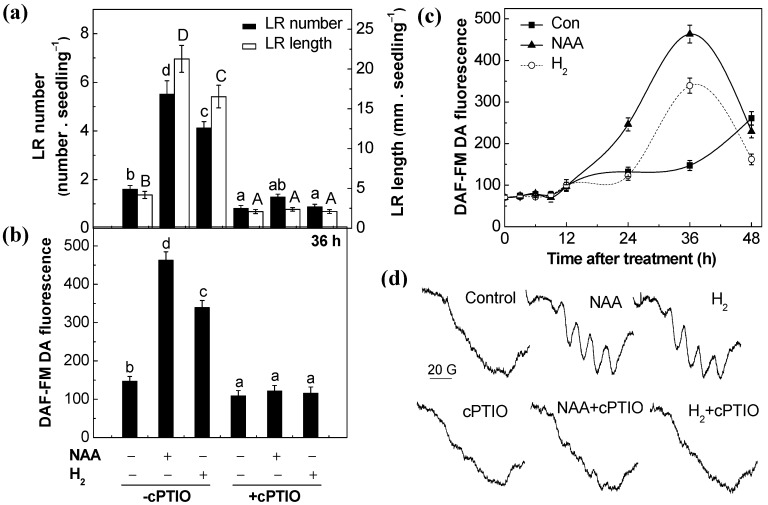
H_2_-induced nitric oxide (NO) production and lateral rooting were sensitive to the removal of NO by 2-4-carboxyphenyl-4,4,5,5-tetramethylimidazoline-1-oxyl-3-oxide (cPTIO), a NO scavenger. 3-day-old tomato seedlings were incubated with solutions containing 200 nM NAA, 0.39 mM H_2_, and 200 µM cPTIO, alone or the combination treatments. (**a**) The number of emerged LRs (>1 mm) per seedling and LR length were calculated after 3-day of treatments. Meanwhile, the NO fluorescence in tomato roots was analyzed by fluorescence probe DAF-FM DA at 36 h (**b**) and over 48 h; (**c**) of treatments, respectively, using laser scanning confocal microscopy (LSCM) (TCS-SP2 system; Leica Lasertechnik GmbH). The DAF-FM DA fluorescence density was analyzed using Leica software. (**d**) The NO signal was also detected by electron paramagnetic resonance (EPR) after being treated for 36 h. Distilled water was used for the control (Con) treatment. Data are the means ± SE of three independent experiments with at least three replicates for each (*n* = 60 for lateral root formation analysis; *n* = 5 for NO detection). Within each set of experiments, bars denoted by the same letter did not differ significantly at *p* < 0.05 level according to Duncan’s multiple range test.

The role of NO in H_2_-induced lateral root formation was further examined by monitoring root organogenesis and NO synthesis in response to applied H_2_ with or without 2-4-carboxyphenyl-4,4,5,5-tetramethylimidazoline-1-oxyl-3-oxide (cPTIO), an effective scavenger of NO. Interestingly, H_2_- and NAA-promoted lateral root formation was greatly impaired in the presence of cPTIO (Figure 3a). Meanwhile, endogenous NO was firstly imaged by laser scanning confocal microscopy (LSCM) with the NO indicator dye 4-amino-5-methylamino-2′,7′-difluorofluorescein diacetate (DAF-FM DA). Significant and progressive increases in NO-induced fluorescence were observed in both NAA- and H_2_-treated tomato seedling roots for 48 h, compared with control (peaking at 36 h; Figure 3b,c and Figure S2). Co-treatment with cPTIO reduced the above NO fluorescence. The above changes in fluorescence were further confirmed by using the highly specific electron paramagnetic resonance (EPR) analysis (Figure 3d). Combined with the data from lateral root formation analysis, the above results demonstrated that NO might act as a downstream component of the complex signaling network inducing lateral root formation triggered by H_2_.

### 2.5. Nitrate Reductase Might Be the NO Enzymatic Source

**Figure 4:**
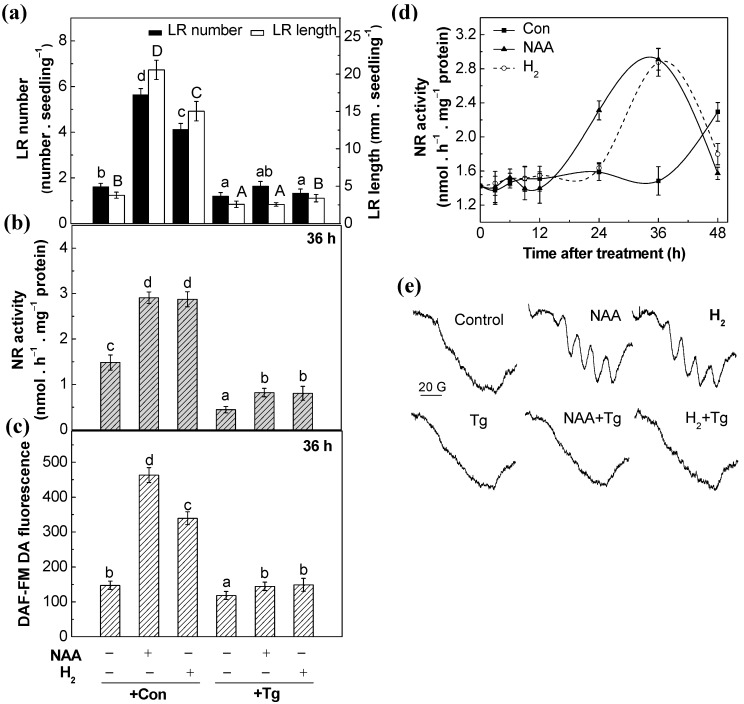
NR might be the enzymatic source of H_2_-triggered NO generation. 3-day-old tomato seedlings were incubated with solutions containing 200 nM NAA, 0.39 mM H_2_, and 20 µM tungstate (Tg), alone or the combination treatments. (**a**) The number of emerged LRs (>1 mm) per seedling and LR length were calculated after 3-day of treatments. Meanwhile, the activity of nitrate reductase (NR) was determined at 36 h (**b**) and over 48 h (**d**) of treatments, respectively; (**c**) The NO fluorescence in tomato roots was analyzed by fluorescence probe DAF-FM DA at 36 h of treatments, using LSCM (TCS-SP2 system; Leica Lasertechnik GmbH). The DAF-FM DA fluorescence density was analyzed using Leica software; (**e**) The NO signal was also detected by EPR after being treated for 36 h. Distilled water was used for the control (Con) treatment. Data are the means ± SE of three independent experiments with at least three replicates for each (*n* = 60 for lateral root formation analysis; *n* = 5 for NR activity and NO detection). Within each set of experiments, bars denoted by the same letter did not differ significantly at *P* < 0.05 level according to Duncan’s multiple range test.

In order to investigate the source(s) of NO synthesis in H_2_-induced lateral root formation, the Nitrate reductase (NR) inhibitor tungstate (Tg) and NOS-like protein inhibitors *N*^G^-nitro-l-arginine methyl ester (NAME) were respectively used. Subsequent results showed that both NAA- and H_2_-induced lateral root length and number, and NR activities were obviously blocked by Tg, while NAME had no such significant effects (Figure 4a,b,d and Figure S3a,b). Consistently, both LSCM and EPR analyses showed that H_2_-induced NO production was more sensitive to Tg (Figure 4c,e). However, there is no significant effect when NAME was applied, at least in our experiments (Figure S3b). Additionally, it is reported that *S*-nitrosoglutathione reductase (GSNOR) can modulate the cellular NO homeostasis [18]. Thus, the potential effect of H_2_ on GSNOR activity after 36 h treatment was studied. We found that H_2_ caused a slight but not significant increase in GSNOR activity (Figure S3c), indicating that GSNOR might not be the enzymatic source of NO generation in H_2_-induced tomato lateral root development. Thereby, our pharmacological and physiological evidence supported that NR might be the main source of NO synthesis during H_2_-induced NO generation and thereafter lateral root formation.

### 2.6. NO Participated in H_2_-Induced Transcript Levels of Cell Cycle Regulatory Genes

**Figure 5:**
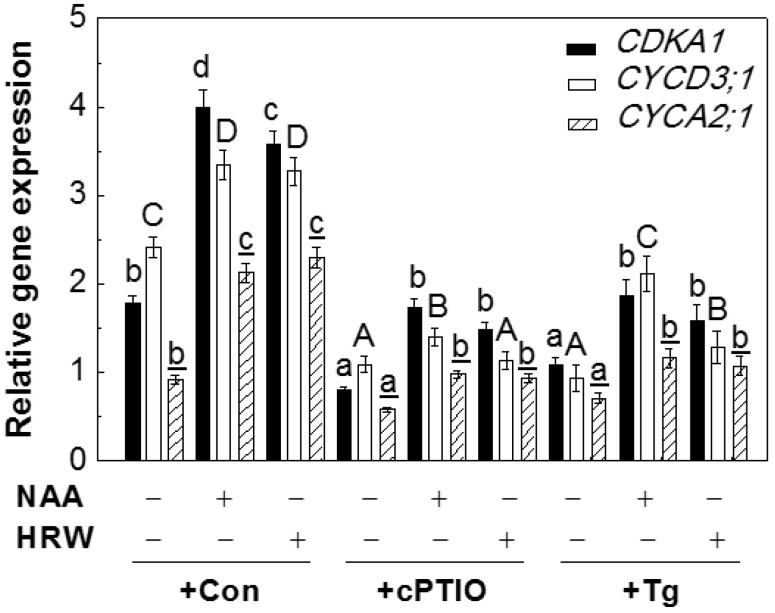
H_2_-induced cell cycle regulatory gene transcripts were sensitive to the removal of NO with its scavenger and NR inhibitor. Three-day-old tomato seedlings were incubated with solutions containing 200 nM NAA, 0.39 mM H_2_, 200 µM cPTIO, and 20 µM tungstate (Tg), alone or the combination treatments for 36 h. The transcripts of tomato *CDKA1*, *CYCD3;1*, and *CYCA2;1* were analyzed using qPCR. Distilled water was used for the control (Con) treatment. Data are the means ± SE of three independent experiments with at least three replicates for each. Within each set of experiments, bars denoted by the same letter did not differ significantly at *p* < 0.05 level according to Duncan’s multiple range test.

To gain insight into the molecular mechanism of H_2_-induced lateral root formation, the changes in the expression profiles of cell cycle regulatory genes (NO-targeted gens responsible for lateral root formation; [1,5]), including *CDKA1*, *CYCD3;1*, and *CYCA2;1*, were analyzed by qPCR. As shown in Figure 5, NAA and H_2_ significantly up-regulated above transcripts, all of which were markedly impaired by the co-treatment with cPTIO or Tg. Our results thus indicated that NR-dependent NO might be involved in H_2_-induced expression of cell cycle regulatory genes responsible for lateral root formation.

### 2.7. Genetic Evidence Supporting the Role of NR-Derived NO in H_2_-Induced Lateral Root Formation

**Figure 6:**
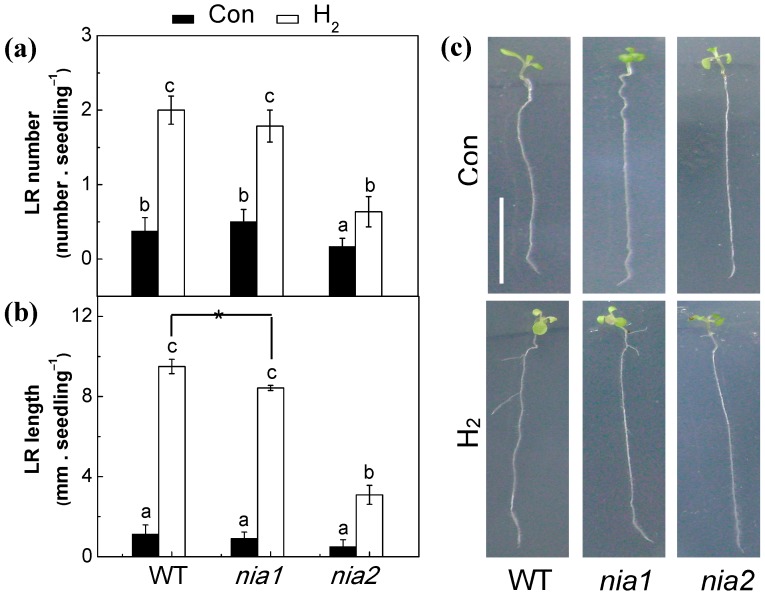
Genetic evidence supporting the involvement of NR in H_2_-induced lateral root formation. 7-day-old Arabidopsis seedlings of wild-type (WT), *nia1*, and *nia2* mutants were treated with semi-strength Murashige and Skoog (MS) medium in the presence or absence of 0.078 mM H_2_. (**a,b**) The number of emerged LRs (>1 mm) per seedling and LR length were calculated after 3-day treatment; (**c**) Representative photographs were then taken. Bar = 1 cm. Treatment without H_2_ was regarded as control (Con). Data are the means ± SE of three independent experiments with at least three replicates for each (*n* = 20). Within each set of experiments, bars denoted by the same letter or labeled with an asterisk did not differ significantly at *p* < 0.05 level according to Duncan’s multiple range test.

To confirm the role of nitric reductase (NR) in H_2_-induced lateral root formation, Arabidopsis *nitric reductase 1* (*nia1*) and *nitric reductase 2* (*nia2*) mutants, both of which are impaired in NO synthesis in plants [19], were further used. As shown in Figure 6a,c, in comparison with the wild-type (WT), *nia2* but not *nia1* mutant under the normal growth conditions, showed a phenotype of impaired lateral root number. Further results revealed that compared to WT, *nia2* (in particularly) and *nia1* mutants were less sensitive to exogenously applied H_2_. The above preliminary genetic evidence supported the idea that NR partially mediates H_2_-induced lateral root formation, at least in Arabidopsis.

## 3. Discussion

Root branching through lateral root formation is an important component of the adaptability of the root system to environment. Although many studies emphasized that the phytohormone auxin acts as a common trigger related to many endogenous and environmental signals governing root organogenesis [1,2,3], knowledge regarding its downstream components in lateral root formation remains limited. The results presented here showed that auxin-induced H_2_ production is associated with lateral root formation, and NR-mediated NO, a well-known important gaseous signaling molecule in plants [20,21], was at least partly, required for the H_2_-triggered lateral root formation promoted by auxin. 

This report showed that the increases in endogenous H_2_ and thereafter NO production are two responses involved in the signaling transduction pathways elicited by auxin in tomato seedling roots. The concentration of H_2_ increased by 43% in tomato seedling roots after 6 h of auxin administration (Figure 1c). Contrasting response was found in NPA-treated seedlings. In the same period, NO concentration was in the basal level, followed by a progressive increase, thus reaching the peak after 36 h of treatment (Figure 3c and Figure S2). We also noticed these two events obviously preceded the beginning of lateral formation. Similar induction in endogenous H_2_ and NO production was also observed in ABA-treated Arabidopsis seedlings [22]. This finding is an interesting event, since a H_2_ increase may have different physiological functions in various experiment systems, when the specific phytohormone(s) was administrated. For example, H_2_-mediated induction of adventitious rooting is correlated with auxin signaling in cucumber explants [17]. The regulation of phytohormone signaling pathway by H_2_ was also preliminarily suggested in plants [23]. Additionally, the number and length of lateral roots and the amount of root hair were increased when the seedlings were inoculated with H_2_-oxidizing bacteria [24].

Subsequent experiments discovered that auxin-induced H_2_ participates in the promotion of lateral root formation (Figure 1a). This conclusion was supported by the following results. First, the increase or decrease in endogenous H_2_ production was observed when NAA or NPA (the auxin transport inhibitor; [6,7]) was individually applied in tomato seedlings, both of which were correlated to the phenotypes of lateral root number and length (induction or inhibition, respectively; Figure 1a,b). Second, NPA-inhibited H_2_ production in vivo and thereafter lateral root formation were obviously alleviated by exogenously applied 0.39 mM H_2_, a concentration confirmed to be effective in our experimental conditions (Figure 2 and Figure S1).

Previous investigation revealed the physiological roles of H_2_ against salt stress [25,26], paraquat-induced oxidative stress [27], and metal toxicity in plants [16,28,29,30]. Interestingly, ABA-triggered H_2_ production was confirmed to participate in the enhancement of drought tolerance in Arabidopsis seedlings [22]. Accordingly, combined with the induction of adventitious rooting in cucumber explants [17], it was further deduced that H_2_ is not only an important plant growth regulator against abiotic stress, but also an inducer of root organogenesis in an auxin-dependent fashion, although auxin-independent pathway also exists [31]. Additionally, H_2_-tgriggered lateral root formation might be a universal event in plants (Table 1).

NO is a signaling molecule involved in many physiological processes during plant development, such as root gravitropism, seed germination, and root organogenesis (including lateral root and adventitious root formation, etc.) [5,7,32,33,34,35]. In response to abiotic stress, NR-mediated NO generation in plants has been demonstrated both in vivo and in vitro [36,37], and it was confirmed to be the downstream component responsible for plant tolerance against environmental stimuli [38]. For example, NR-dependent NO could play a pivotal role in improving the nitrogen acquisition capacity by increasing lateral root initiation and the inorganic nitrogen uptake rate under partial nitrate nutrition in rice [39]. R4, isolated from *Rhizobium leguminosarum* bv. *trifolii*, did not inhibit rice root growth by completing the reduction of NO through to nitrogen gas [40]. Our further pharmacological, physiological, and genetic evidence revealed the causal link between endogenous H_2_ and NO in the induction of lateral root formation.

First, we found that both H_2_ and NAA increased NR activities in tomato seedlings in a time-dependent manner (Figure 4d), and this increased NR activity may account for the induction of endogenous NO levels, which was confirmed by LSCM and ESR analysis (Figure 3c,d and Figure S2). Above increased endogenous NO levels in tomato seedlings, coincident with lateral root formation (Figure 1a), were further reduced by Tg (the NR inhibitor) or cPTIO (the specific scavenger of NO), when applied together with H_2_ or NAA (Figure 3b and Figure 4b,c,e). Meanwhile, H_2_- and NAA-triggered lateral root formation was respectively blocked by the removal of endogenous NO (Figure 3a and Figure 4a). No such responses were observed when the NOS inhibitor, NAME, was used (Figure S3). The possible involvement of NOS-like protein was thus ruled out. Consistently, the requirement of NO in H_2_-promoted adventitious rooting was discovered in cucumber explants as well [41], confirming the central role of NO in root organogenesis. Our previous results revealed that H_2_-stimulated significant induction of NO synthesis was associated with stomatal closure in the wild type of Arabidopsis, which were individually abolished in NR mutants (*nia1/2*) [22]. Together, we deduced that the increased endogenous NO levels mainly resulting from the induction of NR activity were likely to account for the promotion of lateral root formation in response to H_2_. Further data that was subsequently obtained, working with WT and *NR* mutants of Arabidopsis, provided the genetic evidence to support this conclusion (Figure 6).

It was suggested that auxin-triggered NO-mediated cell cycler reactivation occurred by modulating cell cycle regulatory genes in the early lateral root initiation [5,42]. As expected, mimicking the responses of NAA, H_2_ treatment for 36 h could up-regulate the expression of *CDKA1*, *CYCD3;1*, and *CYCA2;1*, all of which were abolished by the removal of endogenous NO (Figure 5). Combined with corresponding phenotypes (Figure 3a and Figure 4a), the above molecular evidence suggested that NO-targeted cell cycle regulatory genes might be involved in the induction of lateral root formation prompted by H_2_.

In conclusion, the above results revealed the involvement of H_2_ in auxin-induced lateral root formation via NO signaling, and provided new insight regarding the use of H_2_ in agriculture.

## 4. Materials and Methods

### 4.1. Chemicals

Unless stated otherwise, all chemicals were obtained from Sigma-Aldrich (St Louis, MO, USA). The chemicals of 1-naphthylacetic acid (NAA), *N*-1-naphthylphthalamic acid (NPA, an inhibitor of auxin transport), 2-(4-carboxyphenyl)-4,4,5,5-tetramethylimidazoline-1-oxyl-3-oxide potassium salt (cPTIO; a scavenger of NO), tungstate (Tg; an inhibitor of NR), and *N*^G^-nitro-l-arginine methyl ester hydrochloride (NAME; a NO synthase-like enzyme inhibitor) were applied [7,8,22]. In this study, the concentrations of the above chemicals were determined in pilot experiments from which the significant responses were observed.

### 4.2. Plant Materials and Growth Conditions

Tomato (*Lycopersicon esculintum*, cv. Jiangshu No. 14), green vegetables (*Brassica chinensis*, cv. Wuqing No. 1), and rice (*Oryza sativa*, cv. Wuyujing No. 7) were obtained from Jiangsu Academy of Agricultural Sciences. Maize (Zea mays, Zhongnuo No. 1) was provided by Chinese Agricultural University. Rapeseed (*Brassica napus* cv. Yangyou No. 6) seeds were kindly supplied by State Key Laboratory of Crop Genetics and Germplasm Enhancement, Nanjing Agricultural University. Seeds were surface-sterilized with 2% NaClO for 10 min, germinated in distilled water at 25 ± 1 °C for 2 days in dark, and seedlings were grown on the illuminating incubator at 25 ± 1 °C with a 200 μmol m^−2^·s^−1^ intensity at 14/10 photoperiod. Afterwards, the selected seedlings were transferred to the indicated chemicals for the indicated time points.

Arabidopsis (*Arabidopsis thaliana*) seeds of *nia1* (CS6936) and *nia2* (CS2355) mutants were obtained from the Arabidopsis Biological Resource Center (available online: http://www.arabidopsis.org/abrc). Seeds were sterilized and rinsed at least three times with sterile water, then cultured in 1/2 Murashige and Skoog (MS, pH 5.8) solid medium containing 1% (*w*/*v*) agar and 1% (*w*/*v*) sucrose. Seeds were kept at 4 °C for 2 days, and then transferred into a growth chamber with 16/8 photoperiod (22/18 °C) with a 200 µmol m^−2^·s^−1^ irradiation for the indicated time points.

Afterwards, photographs were taken, and the number of emerged lateral roots (LRs; >1 mm) per seedling and the length of lateral roots were determined with Image J software. LR primordia (LRP) per seedling were also observed by root squash preparations and quantified by a light microscope (model Stemi 2000-C; Carl Zeiss, Germany; [5]). Additionally, only the lateral root-inducible segments were used for the subsequent biochemical and molecular analyses.

### 4.3. Preparation of H_2_-Rich Water 

Purified H_2_ gas (99.99%, *v*/*v*) generated from a hydrogen gas generator (SHC-300, Saikesaisi Hydrogen Energy Co., Ltd., Ji’nan, China) was bubbled into 1000 mL distilled water at a rate of 150 mL·min^−1^ for 30 min, thus reaching a saturated level. In our experimental conditions, the H_2_ concentration in above saturated H_2_-rich water analyzed by gas chromatography (GC) was about 0.78 mM [25]. The working conditions of GC were optimized as detector temperature of thermal conductivity detector (TCD) at 100 °C, 5 Å molecular sieve as fixed phase, column temperature at 150 °C, and oven temperature at 60 °C. Nitrogen gas was used as carrier gas and air pressure was 0.2 MPa. Afterwards, the corresponding H_2_-rich water was immediately diluted to the required concentrations of H_2_ (0.0078, 0.078, and 0.39 mM).

### 4.4. Measurement of Endogenous H_2_

Endogenous H_2_ was measured by gas chromatography (GC, 5890C, Nanjing Kejie Technology, Ltd, Nanjing, China) [22,27]. Approximately 0.3 g of tomato seedlings were homogenized for 1 min, placed them in a vial, and then 7 mL distilled water, 5 µL octanol and 0.5 mL 5 M sulphuric acid were added. Afterwards, pure nitrogen gas was bubbled in vial to fully displace the air. After it was capped and shaken immediately for 1 min, the vial was heated at 70 °C for 1 h to liberate H_2_ from plant tissues, and allowed to cool at room temperature before the head-space was analyzed.

### 4.5. Detection of Endogenous NO

The endogenous NO level was monitored by a laser confocal scanning microscopy (LCSM) using the specific NO fluorescent probe 4-amino-5-methylamino-2′,7′-difluorofluorescein diacetate (DAF-FM DA) [43,44]. Tomato root samples were chosen at the indicated time points, and loaded with 10 μM DAF-FM DA in 20 mM HEPES/NaOH buffer (pH 7.5) for 30 min, then washed with distilled water for three times. All images were visualized by using LSCM (TCS-SP2 system; Leica Lasertechnik GmbH, Heidelberg, Germany). Five individual samples were randomly selected and measured per treatment. All manipulations were performed at 25 °C. We showed 40-μm-thick sections along Z stack, and the bright-field (BF) images corresponding to the fluorescent images were shown at the bottom right corners. Fluorescence of NO level in roots was quantified based on 20 overlapping confocal planes of 2 µm each using the Leica software.

Endogenous NO production was also quantified by EPR as described previously [22,45,46]. After different treatments, about 0.1 g tomato seedling roots were crushed with a mortar and pestle, then were incubated in 0.3 mL of buffer solution (50 mM HEPES, 1 mM MgCl_2_, 1 mM dithiothreitol, pH 7.6) at room temperature for 2 min. The mixture was added to 0.3 mL of freshly made Fe^2+^(DETC)_2_ solution in dark at room temperature for 2 min. Then, 0.2 mL of ethyl acetate was added, shaken for 3 min and centrifuged at 4 °C (12,000× *g*) for 5 min. EPR was carried out on a Bruker A300 spectrometer (Bruker Instrument, Karlsruhe, Germany) under the following conditions: room temperature; microwave frequency, 9.85 GHz; modulation frequency, 100.00 kHz, microwave power, 63.49 mW.

### 4.6. Determination of Nitrate Reductase (NR) Activity

The NR activity was detected spectrophotometrically at 540 nm according to the previous method [38]. 

### 4.7. RNA Extraction and Real-Time Quantitative RT-PCR (qPCR) Analysis

Total RNA was extracted from roots using Trizol reagent (Invitrogen, Gaithersburg, MD, USA) according to the manufacturer’s instructions. The RNA samples were treated with RNAase-free DNase (TaKaRa Bio, Inc., Dalian, China) to eliminate traces of DNA, and the RNA concentration and quality were detected using the NanoDrop 2000 (Thermo Fisher Scientific, Wilmington, DE, USA). Afterwards, cDNA were synthesized from total RNA (2 μg) using an oligo(dT) primer and M-MLV reverse transcriptase (BioTeke, Beijing, China).

Real-time quantitative RT-PCR (qPCR) was performed using a Mastercycler^®^ ep realplex real-time PCR system (Eppendorf, Hamburg, Germany) with TransStart Top Green qPCR SuperMix (TransGen Biotech, Beijing, China). Using the specific primers (Table S1), the relative expression levels of the corresponding genes were normalized to two internal control genes tomato *CAC* and *TIP41*. The quantification of the relative transcript levels was calculated by using the 2^−ΔΔ*C*t^ method [47,48].

### 4.8. Data Analysis

Where indicated, results are expressed as the means ± SE of at least three independent experiments with at least three replicates for each. Statistical analysis was performed using SPSS 10.0 software. For statistical analysis, either the *t*-test (*p* < 0.05) or one-way analysis of variance (ANOVA) followed by Duncan's multiple range test (*p* < 0.05), was selected where appropriate.

## Figures and Tables

**Table 1:** Exogenous H_2_-induced lateral root formation is universal.

Species	Treatment	LR Number (seedling^−1^)	LR Length (mm seedling^−1^)	LRP Number (seedling^−1^)
*Lycopersicon esculentum*	Con	1.89 ± 0.17	4.03 ± 0.48	1.25 ± 0.25
	0.39 mM H_2_	4.13 ± 0.26 *	15.03 ± 1.30 *	3.38 ± 0.20 *
*Brassica napus*	Con	1.67 ± 0.38	4.00 ± 0.31	1.30 ± 0.11
	0.39 mM H_2_	4.00 ± 0.26 *	15.00 ± 0.51 *	3.48 ± 0.20 *
*Brassica chinensis*	Con	1.74 ± 0.19	4.21 ± 0.43	2.17 ± 0.39
	0.078 mM H_2_	5.14 ± 0.28 *	16.62 ± 1.23 *	3.58 ± 0.12 *
*Oryza sativa*	Con	5.60 ± 1.18	13.00 ± 2.74	1.50 ± 0.24
	0.39 mM H_2_	12.05 ± 1.31 *	38.50 ± 4.78 *	2.92 ± 0.23 *
*Zea mays*	Con	16.67 ± 1.62	40.00 ± 3.85	15.73 ± 0.52
	0.78 mM H_2_	32.41 ± 1.03	89.60 ± 2.48 *	35.33 ± 2.00 *

Con: Control; LR: Lateral root; LRP: Lateral root primordia. Within each set of experiments, Asterisk indicates that mean values are significantly different at the *p* < 0.05 level according to Duncan’s multiple range test.

## Supplementary Materials

Supplementary materials can be found at www.mdpi.com/1422-0067/18/10/2084/s1.

## Abbreviations

LR	lateral root
NAA	1-naphthyl acetic acid
NPA	*N*-1-naphthylphthalamic acid
EPR	electron paramagnetic resonance
cPTIO	2-4-carboxyphenyl-4,4,5,5-tetramethylimidazoline-1-oxyl-3-oxide
NR	nitrate reductase
NO	nitric oxide
NOS	nitric oxide synthetase
H_2_	hydrogen gas
GC	gas chromatography
LSCM	laser scanning confocal microscopy
NAME	*N*^G^-nitro-l-arginine methyl ester hydrochloride
GSNOR	*S*-nitrosoglutathione reductase
Tg	tungstate
