# Supplementary material for: Hydrogen Gas Is Involved in Auxin-Induced Lateral Root Formation by Modulating Nitric Oxide Synthesis
Source: Int J Mol Sci. 2017 Oct 3;18(10):2084. doi: 10.3390/ijms18102084 (PMC5666766; doi:10.3390/ijms18102084)
Supplement: Supplementary file 1 [file ijms-18-02084-s001.pdf]

SUPPORTING INFORMATION

Additional Supporting Information may be found in the online version of this article:

METHODS

1. Measurements of GSNOR Activity

The *S*-nitrosoglutathione reductase (GSNOR) activity was measured by monitoring the decomposition of NADH [1,2]. Oxidation of NADH was determined spectrophotometrically at 340 nm. Seedling root extracts were prepared in 100 mL of 0.05 M HEPES buffer (20% glycerol, 1 mM EDTA, 1 mM benzamidine, 1 mM EGTA,10 mM MgCl2, and 1 mM e-aminocaproic acid, pH 8.0), centrifuged and clarified with a desalting column. Enzyme activity was determined at 25°C by incubating the desalted fraction (10 mL) in 180 mL of 0.1 M phosphate buffer. GSNOR activity was monitored for 1 min after the addition of NADH using an Agilent 8453 UV spectrophotometer. Final NADH decomposition values were normalized against total protein amount. Data are means of three independent experiments.

Table S1. The sequences of primers for qPCR.

Gene name	Gene Accession No.	Primer sequences
CDKA1	Y17225	F: 5’-CACTTGCCTGTCGCCTCCTC-3’
		R: 5’-ACCCCCTCGTCTTCCTGCTC-3’
CYCD3;1	AJ245415	F: 5’-GGTCATTGCTTACTATGGCT-3’
		R: 5’-AAAAGGGGAACTTGGGTCTC-3’
CYCA2;1	AJ243452	F: 5’-CATTAACAAGGGTATGCGAA-3’
		R: 5’-GTCAGGTAAAGAGTGTCCGG-3’
CAC	SGN-U566667	F: 5’-CCTCCGTTGTGATGTAACTGG-3’
		R: 5’-ATTGGTGGAAAGTAACATCATCG-3’
TIP41	SGN-U584254	F: 5’-ATGGAGTTTTTGAGTCTTCTGC-3’
		R: 5’-GCTGCGTTTCTGGCTTAGG-3’

Figure S1.

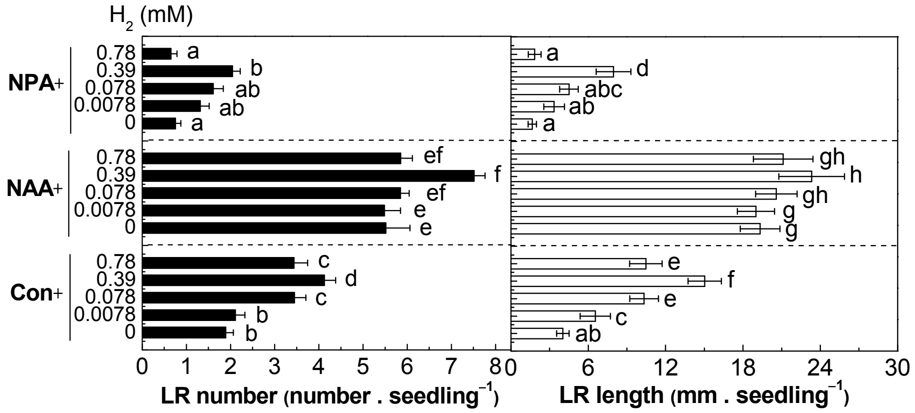

Figure S1. Effects of H_2 on NAA- and NPA-regulated lateral root formation. 3-day-old tomato seedlings were incubated with solutions containing 200 nM NAA, 500 nM NPA, and various concentrations of H_2, alone or the combination treatments. The number of emerged LRs (>1 mm) per seedling and LR length were calculated after 3-day of treatments. Distilled water was used for the control (Con) treatment. Data are the means ± SE of three independent experiments with at least three replicates for each (n=60). Bars denoted by the same letter did not differ significantly at the P<0.05 level according to Duncan's multiple range test.

Figure S2.

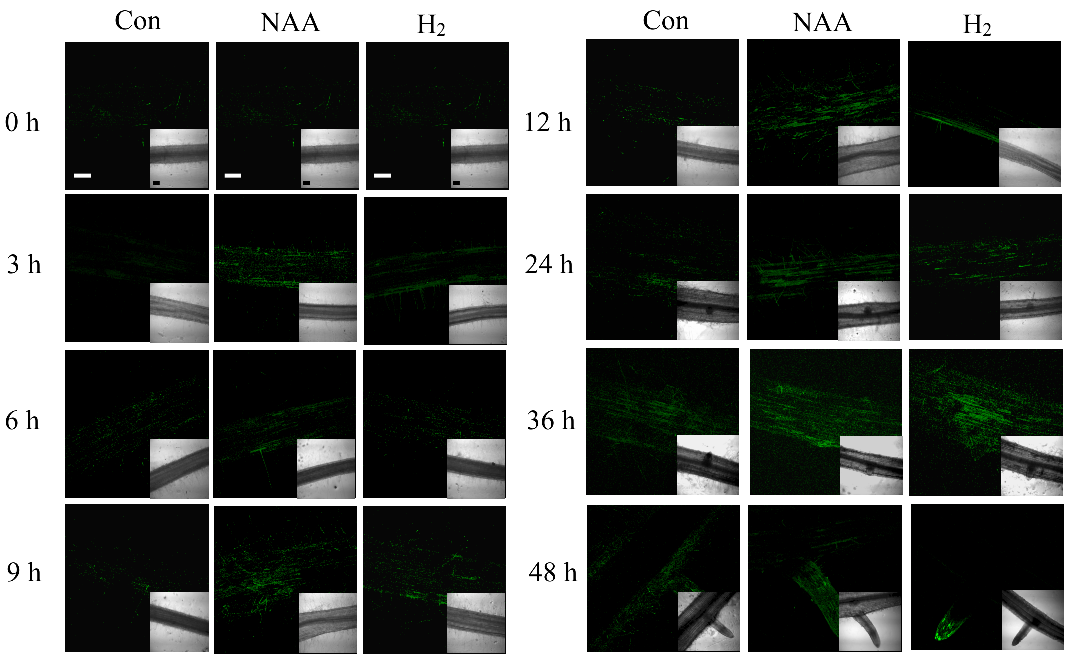

Figure S2. NAA-induced NO production was mimicked by exogenous H_2. 3-day-old tomato seedlings were incubated with solutions containing 200 nM NAA or 0.39 mM H_2. The NO fluorescence in roots was analyzed by fluorescence probe DAF-FM DA at the indicated time points using LSCM (TCS-SP2 system; Leica Lasertechnik GmbH). Distilled water was used for the control (Con) treatment. The DAF-FM DA fluorescence density shown in Fig. 3c, was analyzed using Leica software. Bar=0.2 mm.

Figure S3.

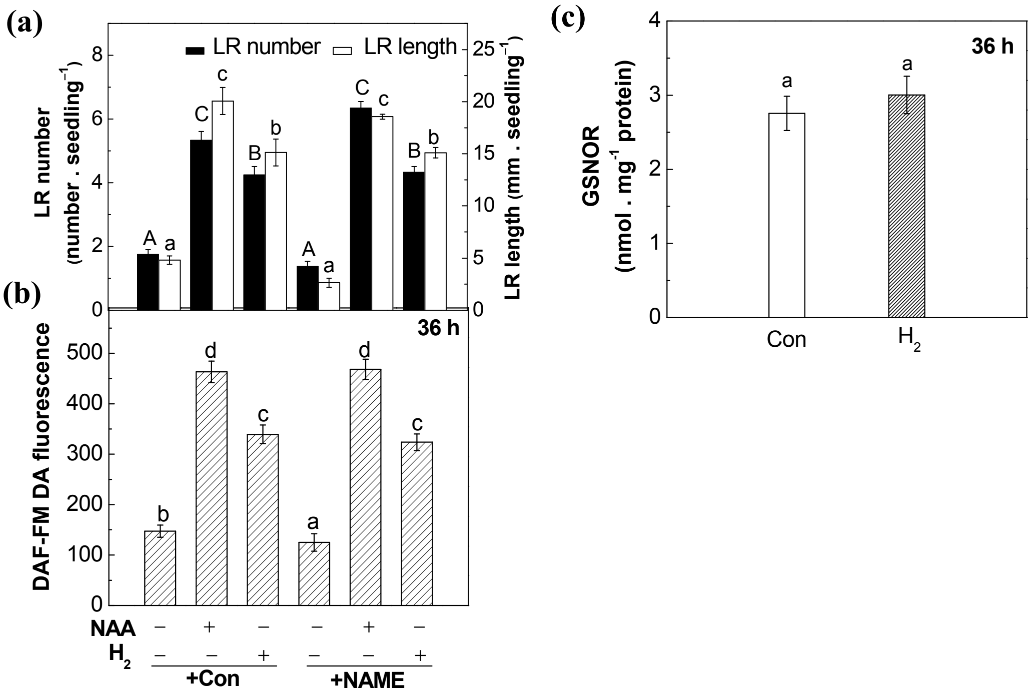

Figure S3. NAME failed to influence H_2-induced lateral root formation and NO fluorescence. 3-day-old tomato seedlings were incubated with solutions containing 200 nM NAA, 0.39 mM H_2, and 200 μM N^G-nitro-L-arginine methyl ester hydrochloride (NAME), alone or the combination treatments. (a) The number of emerged LRs (>1 mm) per seedling and LR length were calculated after 3-day of treatments. (b) After treatment for 36 h, the NO fluorescence in tomato roots was analyzed by fluorescence probe DAF-FM DA using LSCM (TCS-SP2 system; Leica Lasertechnik GmbH). The DAF-FM DA fluorescence density was analyzed using Leica software. (c) The GSNOR activity was not altered by H_2, analyzed after treatment for 36 h. Distilled water was used for the control (Con) treatment. Data are the means ± SE of three independent experiments with at least three replicates for each (n=60 for lateral root formation analysis; n=5 for GSNOR activity and NO

detection). Within each set of experiments, bars denoted by the same letter did not differ significantly at $P<0.05$ level according to Duncan's multiple range test.

REFERENCES

1. Sakamoto, A.; Ueda, M.; Morikawa, H. Arabidopsis glutathione-dependent formaldehyde dehydrogenase is an *S*-nitrosoglutathione reductase. *FEBS Lett.* **2002**, *515*, 20–24.
2. Lee, U.; Wie, C.; Fernandez, B. O.; Feelisch, M.; Vierling, E. Modulation of nitrosative stress by *S*-nitrosoglutathione reductase is critical for thermotolerance and plant growth in Arabidopsis. *The Plant Cell* **2008**, *20*, 786–802.
